# The Child-Parent Relationship During the COVID-19 in Saudi Arabia

**DOI:** 10.7759/cureus.50335

**Published:** 2023-12-11

**Authors:** Najwa N Andajany, Reem Msallam, Karimah M Qutah, Dalia A Alyamani

**Affiliations:** 1 Psychiatry, King Fahad Armed Forces Hospital, Jeddah, SAU; 2 Family Medicine, King Abdulaziz University, Jeddah, SAU; 3 Quality Patient Safety, Horizon Health Network, Fredericton, CAN

**Keywords:** psychology, social distancing, relationship, child, parents, covid-19

## Abstract

Background

The COVID-19 pandemic psychologically affected children and their caregivers. On the other side, parents were adapting to new daily routines for work, education, and self-care in response to the current situation. Therefore, assessing the child-parent relationship during the quarantine period is a crucial issue.

Objective

This study aimed to measure the impact of quarantine during the COVID-19 period on the relationship between children and their parents in the western region of Saudi Arabia, mainly in Jeddah and other nearby cities.

Methods

A cross-sectional survey study was conducted from April to December 2020 in the western region of Saudi Arabia, primarily Jeddah and neighboring cities, during the COVID-19 quarantine and shortly after it was stopped. A validated survey (Child-Parent Relationship Scale (CPRS)) comprising socio-demographic characteristics, conflict, and closeness of the child-parent relationship was distributed to the respondents after being translated from English to Arabic. Statistical analysis was conducted using the Statistical Package for the Social Sciences (IBM SPSS Statistics for Windows, IBM Corp., Version 26.0, Armonk, NY). P-values less than 0.05 were considered statistically significant.

Results

The study included 361 parents with a mean (SD) age of 37.30 (7.77) years old. Most parents were mothers (77.9%, N=279), married (91.3%, N=326), lived with their children (98.3%, N=354), and had three or a lesser number of children (80%, N=287). The average time spent with children was significantly higher after the quarantine (12.96 hours) compared to before the quarantine (8 hours) (p<0.001). The number of hours spent with children before the quarantine was significantly higher for mothers than fathers (8.44 vs. 6.01 hours, respectively, p<0.001). There was a significant association between the mean difference in conflict scores before and after the quarantine and the age of parents (p=0.002), the gender of parents (p<0.001), and marital status (p=0.026). There was a significant correlation between mean differences in closeness scores before and after the quarantine and the gender of parents (p=0.038).

Conclusion

Our findings highlight the complex and varied effects of the pandemic on parent-child relationships. The results emphasize the need for support and interventions to address increased conflict and promote positive relationships between parents and children during challenging times.

## Introduction

The respiratory disease known as COVID-19, which emerged in 2019, is caused by the severe acute respiratory virus syndrome coronavirus 2 (SARS-CoV-2). The first case was in the Wuhan market (China), and the disease spread worldwide after this. In March 2020, the World Health Organization declared COVID-19 a pandemic [1,2]. The first confirmed case in Saudi Arabia was registered on March 2, 2020 [3]. During the pandemic, countries isolated the affected individuals, quarantined the exposed, and applied social distancing measures [4]. Social distancing aims to reduce community interaction and prevent transmission from asymptomatic individuals [4]. The COVID-19 pandemic disrupted normal family routines. Lockdowns and social distancing measures impacted child-parent relationships as well [5,6].

Quarantine psychological effects during the SARS outbreak in Canada revealed a high susceptibility to depression and post-trauma stress disorders [7]. More specifically, mothers with younger children were found to be at a higher risk of developing severe depression and anxiety during the pandemic [8]. Parents in Singapore [9] who experienced a significant impact of COVID-19 reported higher stress levels, harsher parenting, and less closeness with their children. It was also revealed that parents could experience stress disorders during the COVID-19 lockdown [10]. Additionally, children reported higher emotional, self-regulatory, and behavioral problems during the COVID-19 outbreak [11].

Similar to many other countries, Saudi Arabia was influenced by COVID-19 in many aspects, including the economy, healthcare, education, and psychological health [12]. One of these vital aspects is the relationship between parents and their children, which was thoroughly studied in previous research in several countries using a special scale known as the Child-Parent Relationship Scale (CPRS) [12]. However, there is not enough data on the impact of COVID-19 on CPRS in Saudi Arabia, especially in the western region, which has one of the largest populations in the kingdom. Therefore, in this study, we aimed to assess the child-parent relationship in the midst of social distancing in the western region of Saudi Arabia by using the CPRS compared to that before social distancing.

## Materials and methods

Study design

This cross-sectional, observational, population-based, survey-based study was conducted from April to December 2020 in the western region of Saudi Arabia, primarily Jeddah and neighboring cities, during the quarantine of COVID-19 and shortly after the quarantine was stopped.

Study population

The study comprised parents responsible for one or more children and Saudi Arabia residents. Single subjects were excluded from the study population. In addition, married, divorced, and widowed parents were included in the study.

Participants were conveniently recruited from the psychiatry outpatient clinics and telephone services of King Fahad Hospital during quarantine. Additionally, individuals were approached and recruited from Jeddah malls, and snowball sampling techniques were employed by obtaining their telephone numbers.

Sample size

Considering a 90% confidence level and 5% marginal error, the minimum required sample size for this study is 267 subjects.

Data collection

The study survey was designed based on a previously validated survey (CPRS) created by Robert Pianta [12] assessing the strength levels of child-parent relationships. The survey was translated from English to Arabic using the WHO front-to-back translation. In addition, a pilot study was conducted on 14 subjects to test the validity of the questionnaire before conducting the study, and Cronbach's alpha value > 0.7. The questionnaire was distributed as a link to Google form, filled out during phone interviews, or filled out based on face-to-face interviews with the responders.

Statistical analysis

The data was entered through Google Forms and cleaned. The statistical analysis was done using the computer program Statistical Package for the Social Sciences (IBM SPSS Statistics for Windows, IBM Corp., Version 26.0, Armonk, NY). Categorical variables were reported in numbers and percentages. Continuous, distributed variables were reported as the mean and the standard deviation (SD).

The survey consisted of 15 question items, eight items of them test conflicts (questions number 2, 4, 8, 10, 11, 12, 13, and 14) and the other seven items for the closeness items (questions number 1, 3, 5, 6, 7, 9 and 15). The survey questions had a five-point Likert scale answer (strongly disagree, disagree, neutral, agree, and strongly agree). The association between conflict, closeness, and independent variables was conducted using the Mann-Whitney and Kruskal-Wallis tests. The correlation analysis was conducted using the Spearman test. Wilcoxon Signed Rank Test was used to compare two paired groups before and after the quarantine. P-values less than 0.05 were considered statistically significant.

Ethical considerations

Ethical committee approval was received from King Fahad Armed Forces Hospital in Jeddah on 20/06/2020, reference ethical number: REC 363, and verbal informed consent was obtained from the subject before the data collection.

## Results

The study included 361 parents aged from 19 to 64 years with a mean (SD) age of 37.30 (7.77) years. Around half of the parents (52.5%, N=189) live in Jeddah. Most parents were mothers (77.9%, N=279), married (91.3%, N= 326), lived with their children (98.3%, N=354), and had from one to three children (80%, N=287). Parents' children were 54.5% (N=181) boys and 45.5% (N=151) girls. The mean (SD) hours that parents stayed with their children were 8.00 (6.66) hours before social-distancing and 12.96 (7.93) hours after social-distancing, as shown in Table 1.

**Table 1:** Demographic characteristics of the participants The data was represented as mean (standard deviation), median (Interquartile range), and minimum to maximum for numeric data, and number (percentage) for categorical data.

Parameters	Category	N		Percentage	
Gender of parent (N=358)	Father	79		22.1	
	Mother	279		77.9	
What city do you live in? (N=360)	Jeddah	189		52.5	
	Makkah	72		20.0	
	Taif	29		8.1	
	Other	70		19.4	
Number of children (N=359)	One	74		20.6	
	Two	123		34.3	
	Three	90		25.1	
	Four	33		9.2	
	Five	17		4.7	
	Six	16		4.5	
	Seven	3		0.8	
	Eight	1		0.3	
	Nine	2		0.6	
Marital status (N=357)	Married	326		91.3	
	Divorced	26		7.2	
	Widowed	5		1.4	
Gender of the child (N= 332)	Boys	181		54.5	
	Girls	151		45.5	
Do you live with your kids? (N=360)	Yes	354		98.3	
	No	6		1.7	
The hours parent stayed with their children before social distancing. (N=350)	Mean	8.00	SD		6.66
	Median	6	IQR		6
	Min	0	Max		48
The hours parent stayed with their children after social distancing. (N=346)	Mean	12.96	SD		7.93
	Median	12	IQR		17
	Min	0	Max		48

The number of hours spent with children before the quarantine was significantly higher for mothers than fathers (8.44 vs. 6.01 hours, respectively, p<0.001).

Similarly, after the quarantine, the number of spending hours with their children was higher among mothers compared to fathers (13.77 vs. 9.17 hours, respectively, p<0.001).

Moreover, the mean number of hours significantly increased among fathers after quarantine (6.01 hours) compared to before quarantine (9.17 hours) (p<0.001). Similar results were found among mothers; the mean number of hours significantly increased after quarantine (8.44 hours) compared to before quarantine (13.77 hours) (p<0.001).

Additionally, the mean of total number of hours spent with children among parents was significantly higher after the quarantine (12.96) compared to before the quarantine (8) (p<0.001), as shown in Tables 2-3.

**Table 2:** Impact of quarantine on the number of hours spent with children among gender The data was represented as mean (standard deviation), median (Interquartile range), and minimum to maximum for numeric data. The p-value less than 0.05 is considered statistically significant. *Mann-Whitney test

Number of hours spent with children		Fathers	Mothers	P-value*
Before quarantine	Mean (SD)	6.01 (7.08)	8.44 (6.35)	<0.001
	Median (IQR)	4 (5)	6 (7)	
	Min-Max	0-48	1-24	
After quarantine	Mean (SD)	9.17 (7.75)	13.77 (7.59)	<0.001
	Median (IQR)	8 (8)	12 (17)	
	Min-Max	0-48	0-24	

**Table 3:** Impact of quarantine on mothers and fathers regarding the number of hours spent with children The data was represented as mean (standard deviation). A p-value less than 0.05 is considered statistically significant. *Wilcoxon Signed Rank Test

Variables			Number of hours spent with children		p-value*
			Before quarantine	After quarantine	
Gender	Fathers	Mean (SD)	6.01 (7.08)	9.17 (7.75)	<0.001
	Mothers	Mean (SD)	8.44 (6.35)	13.77 (7.59)	<0.001
	Total hours	Mean (SD)	8.00 (6.66)	12.96 (7.93)	<0.001

There was a significant correlation between conflict scores before and after the quarantine (p<0.001). The mean score of conflict after quarantine was 25.70, which was higher than the mean conflict score before quarantine (24.59).

Similarly, there was a significant correlation between closeness scores before and after quarantine (p=0.046). The mean score after quarantine was 31.28, which was slightly higher than the mean closeness score before quarantine (31.13), as shown in Table 4.

**Table 4:** Scores of the conflict and closeness before and after the quarantine The data was represented as mean (standard deviation), median (Interquartile range), and minimum to maximum for numeric data. The correlation was represented as a p-value, significantly less than 0.05. *Wilcoxon Signed Rank Test

Parameters	Category	Mean (SD)	Median (IQR)	Min-Max	p-value*
Conflict scores	Before quarantine (N=352)	24.59 (6.46)	24 (9.75)	12-40	<0.001
	After quarantine (N=351)	25.70 (6.68)	25 (10)	12-40	
Closeness scores	Before quarantine (N=351)	31.13 (3.60)	32 (5)	14-35	0.046
	After quarantine (N=349)	31.28 (3.92)	32 (4)	14-35	

Table 5 represents the difference in conflict scores before and after the quarantine. The parents aged less than or equal to 40 years old had a higher mean difference in conflict scores compared to parents aged more than 40 years (1.83 vs. -0.78, respectively, p=0.002). Mothers had a higher mean difference in conflict scores compared to fathers, with a mean (SD) score of (1.34 vs. 0.22, respectively, p<0.001). Additionally, unmarried parents had a higher mean difference in conflict scores compared to married parents (3.12 vs. 0.89, respectively, p=0.026). However, the city where parents live, the number and gender of children, and whether parents live with their children showed no correlation with the difference in conflict scores before and after the quarantine.

**Table 5:** Correlation between the difference in conflict scores before and after the quarantine with demographic characteristics The data was represented as mean (standard deviation), median (Interquartile range), and minimum to maximum for numeric data. The correlation was represented as a p-value, significantly less than 0.05. *Mann-Whitney test **Kruskal Wallis test

Factors			Difference in conflict score	p-value
Age	≤40 years old	Mean (SD)	1.83 (5.32)	0.002*
		Median (IQR)	1 (4)	
		Min-Max	-19-22	
	>40 years old	Mean (SD)	-0.78 (4.05)	
		Median (IQR)	0 (0)	
		Min-Max	-16-5	
Gender of parent	Fathers	Mean (SD)	0.22 (3.46)	<0.001*
		Median (IQR)	0 (1)	
		Min-Max	-15-8	
	Mothers	Mean (SD)	1.34 (5.21)	
		Median (IQR)	0 (4)	
		Min-Max	-23-22	
What city do you live in?	Jeddah	Mean (SD)	0.52 (4.58)	0.173**
		Median (IQR)	0 (2)	
		Min-Max	-19-17	
	Makkah	Mean (SD)	1.35 (5.80)	
		Median (IQR)	0 (4)	
		Min-Max	-23-19	
	Taif	Mean (SD)	0.93 (2.77)	
		Median (IQR)	0 (2)	
		Min-Max	-5-6	
	Other	Mean (SD)	2.47 (5.26)	
		Median (IQR)	0.5 (5)	
		Min-Max	-8-22	
Number of children	1-3	Mean (SD)	1.27 (5.25)	0.276*
		Median (IQR)	0 (4)	
		Min-Max	-19-22	
	>3	Mean (SD)	0.86 (4.71)	
		Median (IQR)	0 (3.50)	
		Min-Max	-12-12	
Marital status	Married	Mean (SD)	0.89 (4.83)	0.026*
		Median (IQR)	0 (3)	
		Min-Max	-23-22	
	Unmarried	Mean (SD)	3.12 (5.20)	
		Median (IQR)	1.5 (2.75)	
		Min-Max	-2-16	
Gender of the child	Boys	Mean (SD)	1.34 (5.24)	0.153*
		Median (IQR)	0 (4)	
		Min-Max	-23-17	
	Girls	Mean (SD)	0.90 (4.51)	
		Median (IQR)	0 (3)	
		Min-Max	-19-22	
Do you live with your kids?	Yes	Mean (SD)	1.08 (4.93)	0.266*
		Median (IQR)	0 (3)	
		Min-Max	-23-22	
	No	Mean (SD)	2.60 (3.13)	
		Median (IQR)	1 (6)	
		Min-Max	0-6	

Table 6 represents the difference in closeness scores before and after the quarantine. Fathers had a higher mean difference in closeness scores (0.94) compared to mothers who reported a decrease in closeness score after quarantine (-0.004) (p=0.038).

**Table 6:** Correlation between the difference in closeness scores before and after the quarantine with demographic characteristics The data was represented as mean (standard deviation), median (Interquartile range), and minimum to maximum for numeric data. The correlation was represented as a p-value, significantly less than 0.05. *Mann-Whitney test **Kruskal Wallis test

Factors			Difference in Closeness score	p-value
Age	≤40 years old	Mean (SD)	-0.38 (3.57)	0.589*
		Median (IQR)	0 (2)	
		Min-Max	-17-9	
	>40 years old	Mean (SD)	0.67 (2.36)	
		Median (IQR)	0 (1)	
		Min-Max	-2-11	
Gender of parent	Fathers	Mean (SD)	0.94 (2.29)	0.038*
		Median (IQR)	0 (1)	
		Min-Max	-3-11	
	Mothers	Mean (SD)	-0.004 (3.37)	
		Median (IQR)	0 (1.75)	
		Min-Max	-17-17	
What city do you live in?	Jeddah	Mean (SD)	0.31 (3.05)	0.404**
		Median (IQR)	0 (1)	
		Min-Max	-17-11	
	Makkah	Mean (SD)	0.23 (3.95)	
		Median (IQR)	0 (2)	
		Min-Max	-14-17	
	Taif	Mean (SD)	0.28 (2.03)	
		Median (IQR)	0 (0.5)	
		Min-Max	-4-6	
	Other	Mean (SD)	-0.21 (3.20)	
		Median (IQR)	0 (2)	
		Min-Max	-8-9	
Number of children	1-3	Mean (SD)	-0.255 (2.99)	0.156*
		Median (IQR)	0 (2)	
		Min-Max	-10-8	
	>3	Mean (SD)	0.51 (4.75)	
		Median (IQR)	0 (1.5)	
		Min-Max	-17-11	
Marital status	Married	Mean (SD)	0.25 (3.18)	0.227*
		Median (IQR)	0 (1)	
		Min-Max	-17-17	
	Unmarried	Mean (SD)	-0.35 (3.31)	
		Median (IQR)	0 (2.75)	
		Min-Max	-7-6	
Gender of the child	Boys	Mean (SD)	-0.34 (3.23)	0.187*
		Median (IQR)	0 (1)	
		Min-Max	-14-17	
	Girls	Mean (SD)	0.42 (2.80)	
		Median (IQR)	0 (2)	
		Min-Max	-10-8	
Do you live with your kids?	Yes	Mean (SD)	0.18 (3.22)	0.155*
		Median (IQR)	0 (1)	
		Min-Max	-17-17	
	No	Mean (SD)	1 (0.89)	
		Median (IQR)	1 (2)	
		Min-Max	0-2	

However, parents' age and marital status, the city where parents live, the number and gender of children, and whether parents live with their children showed no correlation with the difference in closeness scores before and after the quarantine.

There was a weak negative correlation between age and the mean difference in conflict scores before and after quarantine (r= -0.234, p <0.001). However, the number of kids showed no correlation with the difference in conflict scores.

Moreover, there was no correlation between the difference in closeness scores with age and number of kids, as shown in Tables 7-8.

**Table 7:** Correlation of the difference in conflict scores with age and number of kids The correlation was represented as a p-value, significantly less than 0.05. *Spearman test

Factors		Difference in conflict score	p-value*
Age	Correlation coefficient	-0.234	<0.001
No. of kids	Correlation coefficient	-0.052	0.337

**Table 8:** Correlation of the difference in closeness scores with age and number of kids The correlation was represented as a p-value, significantly less than 0.05. *Spearman test

Factors		Difference in closeness score	p-value*
Age	Correlation coefficient	0.117	0.076
No. of kids	Correlation coefficient	0.076	0.159

## Discussion

During the COVID-19 pandemic, parents and children have likely experienced various changes due to isolation. The isolation, social distancing, and lockdown measures adversely affected individuals' social relationships [13,14]. Our study measured the relationship between parents and their children during the quarantine. It was concluded that the number of hours spent with their children increased significantly after the quarantine among fathers and mothers. Additionally, parents experienced more conflict and more closeness to their children after the quarantine. The age, gender, and marital status of the parents were factors that affected the conflict scores, while the gender of the parents was the only factor that affected the closeness score.

Our results showed a significant increase in hours spent after the quarantine between fathers and mothers, where mothers continue spending more time with their children. Similarly, Parke [15] and Vaterlaus [16] reported that fathers spent more time with their children, but mothers still spent more during the quarantine. In agreement with the Saudi Arabian culture, mothers were more likely to spend significantly longer duration with their children, and this was even more obvious during the lockdown period.

In the present study, the mean conflict scores among mothers were significantly higher than that among fathers which is in accordance with the results by Yaylacı [17]. Moreover, Tarsuslu [18] reported that mothers had higher levels of conflict with their children than fathers, explaining that parents, especially mothers, are often asked by their children to meet their needs. The inability to fulfill such requests may cause them to feel inadequate and exhausted, leading to conflicts.

Our study showed a significant correlation between parents’ age and conflict scores, where parents aged more than 40 years had lower conflict scores after quarantine with their children. Similarly, Tarsuslu [18] revealed significantly higher relationship scores among parents aged 41 to 50 years compared to those aged 31 to 40 years. Moreover, Uzun [19] and Saygı [20] found a weaker relationship between mothers at a younger age and their children. It could be explained by the nature of individuals aged 40 and over who experience biological regression while maintaining mental well-being and social relationships during the transition from mid-adulthood to advanced adulthood [21].

In the present study, there was no significant correlation between children's gender and the child-parent relationship. This aligns with Çakıcı [22] who reported that children's gender did not affect the relationship. In contrast, Uzun [19] and Saygı [20] revealed that mothers have more positive relationships with their daughters.

The COVID-19 pandemic and resulting lockdown have caused a rise in conflicts and challenges for couples [23,24]. Surprisingly, our results revealed a higher conflict between unmarried parents and their children than married parents, which opposed previous studies. A study conducted by Wong [25] reported a higher conflict score of married parents with their children than single parents, which aligns with our study. These results may be because the COVID-19 pandemic has a negative impact on single parents' quality of life, leading to increased conflict with their children during lockdown [26].

Our study experienced more closeness scores regarding child-parent relationships after the quarantine. Similarly, Gambin [27], Tarsuslu [18], and Bilal [28] found that COVID-19 quarantine and social isolation brought closeness between parents and their children. However, Du F [29] and Chung [9] reported worse parent-child closeness during the epidemic. Tan [30] reported more child-parent conflict during the quarantine, which aligns with our study. However, Wong [25] reported less conflict after the quarantine than before. The observed differences can be attributed to the confounding effect of other variables. This might manifest that parents require enough time with their children to have improved connections. They reported less closeness with their children.

One strength of our study is that it examined the impact of quarantine on parent-child relationships during the COVID-19 pandemic. The study provided valuable insights into how isolation, social distancing, and lockdown measures affected the dynamics between parents and their children. By measuring the number of hours spent with children, conflict levels, and closeness scores before and after quarantine, we were able to capture changes in parent-child relationships during this challenging period.

On the other hand, the findings of our study have several implications. Firstly, the significant increase in the number of hours parents spend with their children after the quarantine highlights the importance of quality time and parental involvement in children's lives. This has implications for promoting positive parent-child interactions and fostering healthy development in children. Secondly, the higher conflict scores observed after the quarantine indicate the potential challenges and strains experienced by parents during this period. Understanding the factors contributing to conflicts, such as parental age and gender, can help inform interventions and support systems to alleviate these difficulties and enhance family well-being. Thirdly, the increased closeness scores post-quarantine suggest that despite the challenges, the pandemic also provided an opportunity for parents to develop stronger bonds with their children. This finding underscores the resilience and adaptability of families in coping with adverse circumstances.

However, the small sample size limits the validity of our findings. In addition, other factors not examined in this study, such as economic factors (income level, employment status, and financial stress), parental mental health, and children's behaviors, may affect child-parent relationships. Therefore, further studies are highly needed to improve the relevance of these findings by a larger sample size and investigating all possible variables that could affect the child-parent relationship. Socio-demographic variables, physical health of the parents, financial status, children’s daily routines, mental health, and lifestyle need to be investigated to identify factors affecting child-parent relationships.

## Conclusions

Family is the most important institution in the socialization of any child. Parental positive interaction supports better development of the child. After the quarantine, a higher number of hours parents stayed with their children, more conflict after the quarantine, and more closeness to their children was observed. The parents' age, gender, and marital status affected conflict scores, while only parental gender affected closeness. Our findings highlight the complex and varied effects of the pandemic on parent-child relationships. The results emphasize the need for support and interventions to address increased conflict and promote positive relationships between parents and children during challenging times. Providing adequate time for parent-child interactions and considering individual and cultural factors can contribute to nurturing healthier and more harmonious relationships.
